# *Helicobacter pylori* infection can affect energy modulating hormones and body weight in germ free mice

**DOI:** 10.1038/srep08731

**Published:** 2015-03-04

**Authors:** Yalda Khosravi, Shih Wee Seow, Arlaine Anne Amoyo, Kher Hsin Chiow, Tuan Lin Tan, Whye Yen Wong, Qian Hui Poh, Ignatius Mario Doli Sentosa, Ralph M. Bunte, Sven Pettersson, Mun Fai Loke, Jamuna Vadivelu

**Affiliations:** 1 Department of Medical Microbiology, Faculty of Medicine, University of Malaya, Kuala Lumpur, Malaysia; 2 National Cancer Centre, Singapore, Singapore; 3 School of Chemical and Life Sciences, Singapore Polytechnic, Singapore; 4 Duke-NUS Graduate Medical School, Singapore; 5 Department of Microbiology, Tumor and Cell Biology (MTC), Karolinska Institutet, Stockholm, Sweden; 6 LKC School of Medicine, Nanyang Technological University, Singapore, Singapore; 7 SCELSE Microbiome Centre, Nanyang Technological University, Singapore, Singapore

## Abstract

*Helicobacter pylori*, is an invariably commensal resident of the gut microbiome associated with gastric ulcer in adults. In addition, these patients also suffered from a low grade inflammation that activates the immune system and thus increased shunting of energy to host defense mechanisms. To assess whether a *H. pylori* infection could affect growth in early life, we determined the expression levels of selected metabolic gut hormones in germ free (GF) and specific pathogen-free (SPF) mice with and without the presence of *H. pylori*. Despite *H. pylori*-infected (SPFH) mice display alteration in host metabolism (elevated levels of leptin, insulin and peptide YY) compared to non-infected SPF mice, their growth curves remained the same. SPFH mice also displayed increased level of eotaxin-1. Interestingly, GF mice infected with *H. pylori* (GFH) also displayed increased levels of ghrelin and PYY. However, in contrast to SPFH mice, GFH showed reduced weight gain and malnutrition. These preliminary findings show that exposure to *H. pylori* alters host metabolism early in life; but the commensal microbiota in SPF mice can attenuate the growth retarding effect from *H. pylori* observed in GF mice. Further investigations of possible additional side effects of *H. pylori* are highly warranted.

The largest human microbiome is the digestive tract, more accurately the large intestine. It harbors approximately 100 trillion (10^14^) bacterial cells and more than a 100 times the quantity/number of genes of the human genome^1^. We acquired the gut microbiota at the time of birth or immediately after birth and its composition depends on many factors, including the genetic background, environment and diet^1^,^2^. The microbiome is usually heterogeneous during the first day of life, and after the first week, a stable bacterial flora will be established^3^. The composition and complexity of the microbiome can be affected by physiological changes, such as aging and pregnancy^3^,^4^. Antibiotic treatment and metabolic, immunological or infectious diseases will also change the gut microbiota^5^. The gut microbiota plays a significant role in many vital functions, such as energy harvest from the diet and energy storage^4^,^5^, development and regulation of the gut-associated mucosal immune system^3^, regulation of the central nervous system^6^, detoxification of xenobiotics and carcinogens, and protection against colonization by pathogens^7^. Alteration of fecal and intestinal mucosal microbiome may also be a cause of inflammatory bowel disease, obesity and metabolic syndrome. A recent study comparing germ-free (GF) mice (absence of normal gut microbiota) and their specific pathogen free (SPF) counterparts (with normal gut microbiota) showed that the absence of normal gut microbiota could affect the mice's behavior and the expression of genes that regulate motor control and anxiety^8^. Interestingly, in the same study, the authors also demonstrated that GF mice inoculated with normal gut microbiota early in life displayed similar characteristics as SPF mice. Thus, they postulated that normal gut microbiota might be responsible for modulating brain development during early stages of life.

The ancient gastric pathogen, *Helicobacter pylori*, is a key member of the human gastric microbiome. It co-evolved with the human race and our association with *H. pylori* could be traced as early back as 60,000 years ago^9^. Furthermore, it resides in the stomach of more than half of the global population regardless of ethnicity and geographical region^10^. Many acquired the bacteria during early childhood via intra-familial transmission and colonization is life-long unless eradicated through medical intervention^9^. Although most carriers remain asymptomatic, about 20% of them eventually develop gastro-duodenal diseases (such as chronic gastritis, peptic ulcer and gastric adenocarcinoma) in the later stages of life^11^. Many factors, including *H. pylori*, gut microbiota, the host and the environment, may contribute to the development of *H. pylori*-associated diseases.

In this study, we aimed to investigate the interplay between *H. pylori* and normal gut microbiota during early stages of life using GF and SPF mice models. In order to understand the influences of *H. pylori* and normal gut microbiota on the development of metabolic and immune systems, we assessed the changes of gut metabolic hormones and cytokines/chemokines in response to *H. pylori* colonization in both GFH and SPFH mice.

## Results and Discussion

We recovered *H. pylori* from inoculated SPFH and GFH mice at 2-, 8- and 16-weeks post-colonization. This showed that *H. pylori* strain 298 successfully colonized the stomach of the mice inoculated with this organism. On the other hand, none of the SPF and GF mice was positive for *H. pylori*.

**Figure 1:**
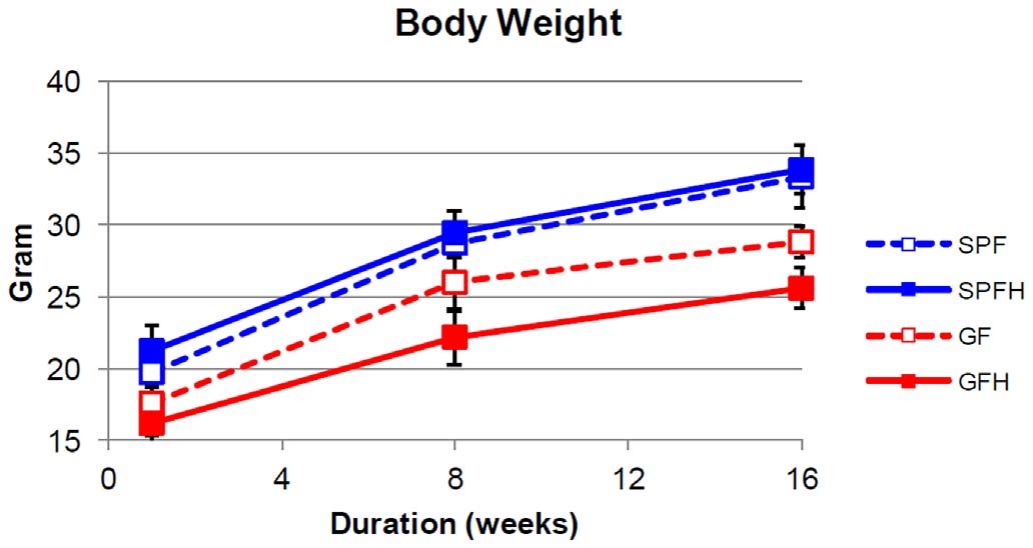
Mean body mass of mice. GF mice weighed significantly less compared to their SPF counterparts (p < 0.01). At the end of 16 weeks, neither SPF nor SPFH mice suffered from any significant weight loss (p > 0.05). However, GFH mice suffered from significant weight loss compared to GF mice (p < 0.01).

In this study, we measured the weight of the control SPF, *H. pylori*-colonized SPFH, control GF and *H. pylori*-colonized GFH mice at 2-, 8- and 16-weeks post-colonization (Figure 1). At the end of 16 weeks, neither SPF nor SPFH mice suffered from any significant weight loss (p > 0.05). However, GFH mice suffered from significant retarded weight gain compared to GF mice (p < 0.01). This showed that in the absence of normal gut microbiota, *H. pylori* colonization caused significant reduced weight gain. GF mice weighed significantly less compared to their SPF counterparts (p < 0.01), which is consistent with previous reports from other research groups^12^,^13^. Earlier studies have shown that germ-free mice needed 30% more calories compared to conventional mice in order to keep a similar body mass^12^. Similarly, another study demonstrated that germ-free mice ate more but had considerably less body fat than conventional mice^13^. In the same study, germ-free mice that were inoculated with microbiota harvested from the distal intestine (cecum) of conventionally raised mice gained 60% of body fat. Indeed, normal gut microbiota plays an important role in determining energy harvest from the diet and energy storage in its host. However, the role of *H. pylori* and its interaction with normal gut microbiota have not been studied.

### Gut Microbiota and *H. pylori* influenced Leptin and Ghrelin Levels

**Figure 2:**
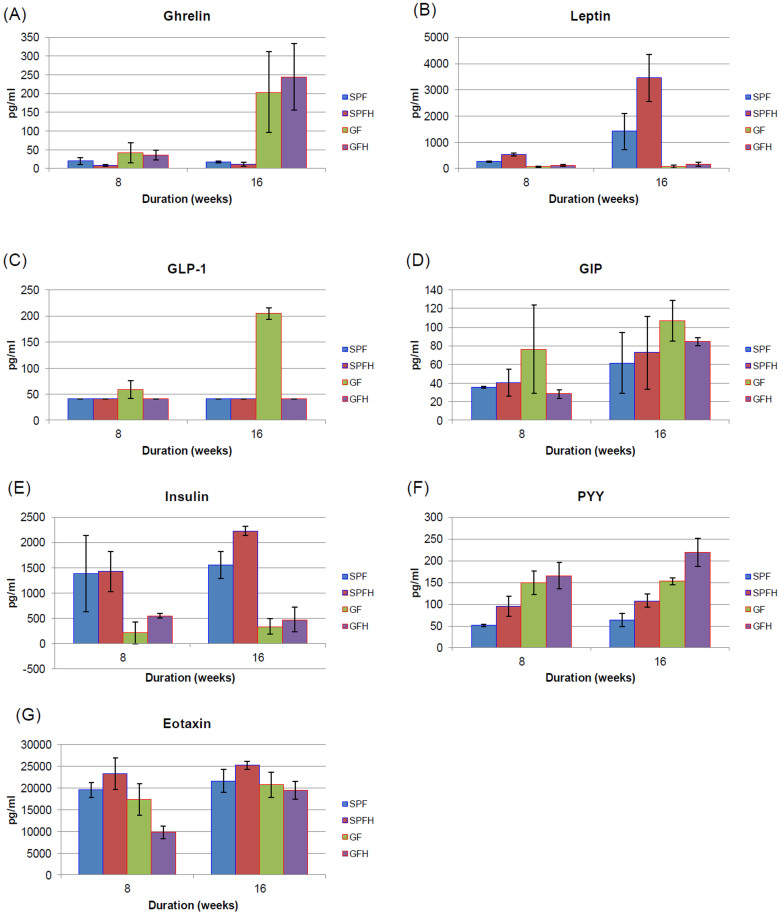
Fasting levels of hormones related to energy homeostasis and eotaxin-1. (A) GF and GFH mice had significantly higher ghrelin level by 16 weeks into the experiment than SPF and SPFH mice (p < 0.01). (B) GF mice had significantly lower leptin level than SPF mice (p < 0.05). Both 8- and 16-weeks SPFH mice had significantly increased leptin production compared to SPF mice (p < 0.01). (C) GLP-1 was significantly lower in SPF, SPFH and GFH mice in comparison to GF mice (p < 0.05) at 8- and 16-weeks post-colonization. (D) Increase of GIP in GF mice was not statistically significant ((p < 0.05). (E) Fasting insulin level was significantly higher in 16-week SPF mice than in GF mice (p < 0.01). SPFH mice showed increased insulin production compared to SPF mice (p < 0.05). (F) PYY level was significantly suppressed in SPF mice by 8 weeks and onwards into the experiment (p < 0.01) but were significantly higher in SPFH and GFH mice compared to SPF (p < 0.05) and GF (p < 0.01) mice. Fasting PYY level was highest among GFH mice followed by those of GF mice. (G) The level of chemokine, eotaxin-1, was found to be highest in SPFH mice (p < 0.01) at 16 weeks post-colonization (Figure 2G).

Leptin and ghrelin, along with many other hormones, participate in the complex process of energy homeostasis. Ghrelin (“hunger hormone”) was originally isolated from the stomach, but ghrelin has also been identified in other peripheral tissues, such as the pancreas, ovary and adrenal cortex^14^. In the brain, ghrelin-producing neurons have been identified in the pituitary and the hypothalamus glands^15^. In the stomach and small intestine, ghrelin is released into circulation as acyl-ghrelin^16^. Leptin (“satiety hormone”) is produced mainly in the adipocytes of white adipose tissue. Brown adipose tissue, syncytiotrophoblasts in the placenta, ovaries, skeletal muscle, stomach (the lower part of the fundic glands), mammary epithelial cells, bone marrow, pituitary, liver, gastric chief cells and P/D1 cells also produce leptin^17^. Both ghrelin and leptin receptors are localized on the same brain cells (mainly in the hypothalamus), therefore these cells receive competing signals of satiety and hunger^18^. In this study, we showed that fasting plasma samples of GF and GFH mice had significantly higher ghrelin level by 16 weeks into the experiment than SPF and SPFH mice (p < 0.01), whether or not they were *H. pylori* colonized (Figure 2A). Therefore, normal gut microbiota, but not *H. pylori*, had significant influence on mice's ghrelin level. On the contrary, GF mice had significantly lower leptin level than SPF mice (p < 0.05) (Figure 2B). Normal gut microbiota, independent of *H. pylori*, was sufficient to up-regulate leptin production and down-regulate ghrelin secretion.

In addition, *H. pylori* colonization caused significantly increased leptin production in SPFH mice compared to SPF mice (p < 0.01), which became obvious by 8-week post-colonization (Figure 2B). In contrast, *H. pylori* did not cause higher leptin secretion in GFH mice compared to GF mice in the absence of normal gut microbiota. Thus, *H. pylori* and normal gut microbiota resulted in up-regulated leptin but not ghrelin production in SPFH mice.

### Normal Gut Microbiota and *H. pylori* induced Insulin Production

Gastric inhibitory polypeptide (GIP) and glucagon-like peptide-1 (GLP-1) belong to a class of molecules referred as incretins. L-cells in the ileum secrete GLP-1 postprandial^19^ while K-cells, which are found in the mucosa of the duodenum and the jejunum, synthesize GIP^20^. The incretins play an important role in stimulating insulin production in response to the presence of nutrients in the intestine. Pancreatic ß-islet cells produce insulin, which is the most essential hormone controlling glucose and energy homeostasis^21^. Normal gut microbiome could contribute nutrients and energy to the host via the fermentation of non-digestible dietary components, thereby directly stimulating insulin production in the host. Actions of insulin on cells include: (1) prompting the uptake and storage of glucose in liver and muscle cells in the form of glycogen, (2) prompting fat cells to take in blood lipids and converting to triglycerides, (3) prompting adipose tissue to synthesize fats (i.e., triglycerides) from fatty acid esters, (4) prompting cells to absorb circulating amino acids, and (5) decreasing protein breakdown^22^. Insulin has a stimulatory effect on leptin, which is produced by fat cells^22^. In turn, leptin acts on receptors in the hypothalamus, where it inhibits hunger by (1) counteracting the effects of neuropeptide Y, (2) counteracting the effects of anandamide, and (3) promoting the synthesis of α-melanocyte-stimulating hormone (α-MSH)^23^. Leptin also interacts with amylin to reduce gastric emptying and creating a feeling of fullness^24^. They work together to maintain energy homeostasis.

We showed that GLP-1 was significantly lower in SPF, SPFH and GFH mice in comparison to GF mice (p < 0.05) when measured at 8- and 16-weeks post-colonization (Figure 2C). A similar trend was also observed for another member of the incretin family, GIP. However, the increase of GIP in GF mice was not statistically significant (Figure 2D). This suggested that microbial colonization (*H. pylori* or normal gut microbiota alone or together) down-regulated the expression of host GLP-1 or accelerated the degradation of incretins. However, insulin level in our study did not correlate with levels of the incretins. Fasting plasma insulin was low in GF mice despite higher levels of incretins (Figure 2E). Fasting insulin level was significantly higher in 16-week SPF mice than in GF mice (p < 0.01), suggesting that normal gut microbiota could influence circulating insulin level. Furthermore, SPFH mice showed increased insulin production compared to SPF mice (p < 0.05). Interestingly, leptin and insulin levels in plasma of GFH mice were not altered.

### PYY induced by *H. pylori* but suppressed by Normal Gut Microbiota

Peptide YY (PYY) is found in L cells in the mucosa of gastrointestinal tract, especially in the ileum and colon. In addition, a small amount of PYY, about 1–10%, is found in the esophagus, stomach, duodenum and jejunum^25^. Like leptin, PYY exerts its action through NPY receptors to slow the gastric emptying that increases the efficiency of digestion and nutrient absorption after a meal^26^. Interestingly, the presence of normal gut microbiota in SPF mice appeared to suppressed fasting PYY level significantly by 8 weeks and onwards into the experiment (p < 0.01) (Figure 2F). On the other hand, *H. pylori* appeared to up-regulate PYY production, such that fasting level was significantly higher in SPFH and GFH mice compared to SPF (p < 0.05) and GF (p < 0.01) mice.

Fasting PYY level was highest among GFH mice followed by those of GF mice (Figure 2F). High PYY is usually associated with the consumption of protein and was observed in experimental subjects to reduce hunger and promote weight loss^27^. This would help to explain the weight-loss experienced with high-protein diets. Therefore, it was not surprising that in this study, we found an inverse correlation between PYY level and the degree of weight loss. Since high level PYY was not linked to high protein diets, it might suggest increased endogenous protein degradation and therefore a loss of body mass. Another factor that might be indirectly linked to the retarded weight gain observed in GF mice is ghrelin, which represents the host's response by decreasing energy expenditure. Individuals with anorexia nervosa have high plasma level of ghrelin compared to individuals who are constitutionally thin as well as normal-weight controls^28^. Together, high ghrelin and high PYY could account for the retarded weight gain in GF and GFH mice and the animals that might be in a state of malnutrition.

The impact of *H. pylori* on the composition and function of the normal gut microbiome has not been fully elucidated. Nevertheless, in this study, our preliminary results demonstrated that *H. pylori* may alter the energy homeostasis of its host, and that the normal gut microbiome could make a difference on the effect of *H. pylori* on its host.

### Gut Metabolic Hormones and Body Mass

A meta-analysis reporting on *H. pylori* and obesity prevalence rates in random population samples of more than 100 human subjects and discovered there was an inverse correlation between prevalence of the bacteria and rate of overweight/obesity in developed countries^29^. Earlier, circulating meal-associated leptin and ghrelin levels were shown to change after *H. pylori* eradication in human volunteers with an associated increase in body mass intex (BMI) providing evidence that *H. pylori* colonization might regulate ghrelin and leptin levels, which affects body morphometry^30^. In this study, *H. pylori* colonization in young GF mice was shown to result in reduced gain in body mass.

SPFH mice had significantly higher fasting leptin, insulin and PYY than SPF mice. Despite the differences in hormonal levels, SPFH mice had normal body mass. We postulate that *H. pylori*-associated up-regulation of PYY stimulated the host to produce more insulin and leptin in response, thus sustaining normal body mass. However, the long-term implications of higher fasting PYY, insulin and leptin remain to be determined. On the other hand, GF and GFH mice had higher ghrelin, lower leptin and lower insulin than SPF and SPFH mice. In addition, 16-week GFH mice had higher PYY level than GF mice. All these hormonal differences between GF and GFH mice in comparison to SPF and SPFH mice might be linked to their body masses. Interestingly, the effect of *H. pylori* on body mass was most obvious in GFH mice in the absence of normal gut microbiota.

The relationship between body mass and different gut metabolic hormones/eotaxin-1 was determined (Table 1). Leptin and PYY levels were shown to be significantly related (p < 0.05) to body mass in both 8-week and 16-week mice in direct and inverse relationships respectively. However, ghrelin and insulin levels were not related to body mass at 8 weeks (p ≥ 0.05) but were significantly inversely and directly correlated respectively in 16-week mice (p < 0.05). Surprisingly, eotaxin-1 levels correlated significantly with body mass initially at 8 weeks (p < 0.05) but not at the later 16 weeks (p ≥ 0.05). These results suggest that both leptin and PYY influenced body mass at an early stage and continue to do so at later stage. On the other hand, ghrelin and insulin, which became related to body mass only at a later stage, might be in responds to the initial changes in leptin and PYY levels. Eotaxin-1 might be an indirectly respond to the initial change in body mass but still have an important influence on the early development in these young mice.

In our study, *H. pylori* infection in GFH mice was associated with high plasma PYY level. This may signify high energy expenditure and high rate of metabolism in these mice, which translate to rapid loss of body mass. However, the presence of normal gut microbiota in SPFH mice might provide buffering against the direct adverse impact of *H. pylori* on body mass. On the other hand, high level of leptin in SPFH mice might suppress food intake and thereby preventing the mice from becoming obese over long term. Results on SPFH mice from this study is consistent with the observation in human subjects that *H. pylori* eradication resulted in increased BMI^30^. Thus, *H. pylori* regulating metabolic hormones may play a role in keeping obesity at bay.

### Possible Impacts of Gut Microbiota and *H. pylori* on Early Development

Heijtz *et al*. reported that the same GF mice used in this study demonstrated increased motor activity and reduced anxiety compared to SPF mice^8^. This correlated with altered expression of genes regulating motor control and anxiety-like behavior. The same report also demonstrated that GF mice exposed to normal gut microbiota early in life had similar characteristics to those of SPF mice. This observation might be related to impaired nutritional absorption in the GF mice in the absence of normal gut microbiota, which led to malnutrition and weight loss. It is well known that the nutritional level has a direct impact on early developmental stages (including brain and mentor development). Furthermore, ghrelin knockout mice had been shown to have increased anxiety in response to a variety of stressors, such as acute restraint stress and social stress, in experimental settings^31^. Higher ghrelin level in GF mice might also explain the altered behavior in GF mice reported by Heijtz *et al*.

Increased eotaxin-1 level in blood plasma is also associated with aging in mice and humans^32^. It has been demonstrated that exposing young mice to eotaxin-1 or the blood plasma of older mice decreased their neurogenesis and cognitive performance in behavioral tasks, which are dependent on neurogenesis in the hippocampus^32^. Interestingly, a large population cohort retrospective study in Denmark has established a correlation between chronic *H. pylori* and/or gastritis and Parkinson's disease^33^. The level of chemokine, eotaxin-1, was found to be highest in SPFH mice (p < 0.01) at 16-week post-colonization (Figure 2G). Eotaxin-1 is one of the chemokines that mediates eosinophil migration, a prominent component of *H. pylori*–induced gastritis^34^. Thus, high level of eotaxin-1 in SPFH mice led us to hypothesize that these chemokines served as early indication of inflammation and the interplay between *H. pylori* and normal gut microbiota may intensify chronic inflammation. Therefore, it is possible that *H. pylori* exposure during early developmental stages will have long-term implication on brain development. Unfortunately, we did not carry out any behavioral study or motor control tests in this study for confirmation.

**Figure 3:**
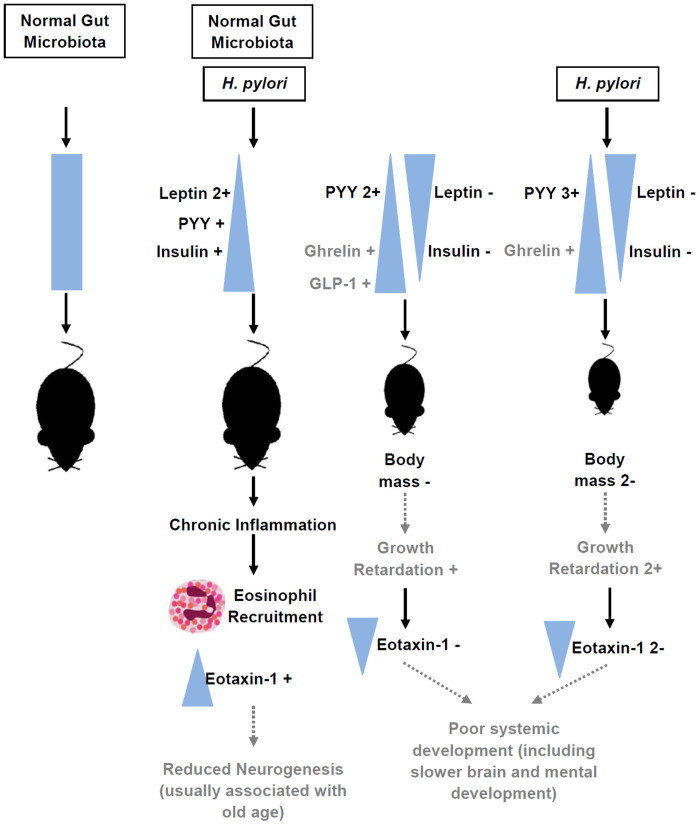
Diagrammatic representation of the effect of normal gut microbiota and *H. pylori* in mice. Normal gut microbiota plays an important role regulation of gut metabolic hormone homeostasis that affects the development of normal body mass and brain. In addition, normal gut microbiota also influences the outcome of *H. pylori* colonization by affecting the gut metabolic hormone changes and inflammation induced by *H. pylori*.

In conclusion, this study in SPF and GF mice with and without *H. pylori* imply that the gastric pathogen, *H. pylori*, interacts with normal gut microbiota, which alters metabolic and inflammatory pathways on SPFH mice compared to SPF mice (Figure 3). Our results also suggest that there is an ongoing crosstalk between *H. pylori* and the normal gut microbiota which associates with gut inflammation. Previous data have established causality between *H. pylori* and gastric ulcer and stomach cancer. The data presented in this study, connecting *H. pylori* to crosstalk with the normal gut microbiota, imply that the onset of *H. pylori* mediated diseases in humans is more complex. Additional experiments are therefore highly warranted, in particular in early life.

## Methods

### *H. pylori* Strain and Inoculum Preparation

The C57BL/6 mice-adapted *H. pylori* strain, 298, was used in this study. The genome of this strain has been fully sequenced^35^. Briefly, *H. pylori* was cultured in Brain Heart Infusion Broth supplemented with 0.4% yeast extract and 1% ß-cyclodextran (BHYC) for 4 days at 37°C in a humidified 10% CO_2_ incubator. The bacteria count was adjusted to the concentration of 10^9^ bacteria per ml to be used for animal inoculation.

### Animals and Inoculation

Male SPF and GF C57BL/6 mice of 4–6 weeks old were used for experiments. Male mice were used because they have been reported to show a higher incidence of intestinal-type gastric carcinoma than females^36^. All mice (SPF and GF) were maintained in sterile plastic isolators at the National Cancer Centre's germ-free facility (NCC) housed in the SingHealth Experimental Medicine Centre. Animals were maintained on autoclaved R36 Lactamin Chow (Lactamin, Sweden) and kept under 12-h light cycles condition. The animals were divided into four groups of 12 (SPF, SPFH, GF and GFH). SPF and GF mice for inoculation with *H. pylori* were fasted overnight and fed intragastrically with 0.1 ml of bacterial culture three times with a 1-day interval in between. Control animals were inoculated with sterile BHYC media in the same way. Subsequently, mice were sacrificed 2 weeks (N = 3 per group), 8 weeks (N = 5) and 16 weeks (N = 5) post-colonization. Mouse gastrics were collected for *H. pylori* culturing, and plasma was used for hormone and cytokine analysis.

### Ethics Statements

All experimental procedures were approved and carried out in accordance to the regulations and guidelines of the SingHealth Institutional Animal Care and Use Committee (IACUC; Application #2011/SHS/680) and National Cancer Centre Singapore Research Biosafety Committee/SingHealth Institutional Biosafety Committee (IBS; Application #010/RBC/2011).

### Plasma Sample Collection

Blood was drawn into pre-chilled EDTA-coated tubes and mixed gently by inversion. Blood was transferred into a chilled Eppendorf tube containing 2 M 4-(2-Aminoethyl) benzenesulfonyl fluoride hydrochloride (AEBSF, Sigma-Aldrich, Buchs, Switzerland) to a final concentration of 0.2 M and mixed gently. The tube was centrifuged at 3,000 × g for 3 min (4°C). The resultant plasma was added to a cryotube containing 1N HCl to give a final concentration of 0.1N and was stored at −80°C until analysis.

Culturing *H. pylori* from mouse gastric stomach was homogenized and inoculated onto chocolate agar plates supplemented with 7% lysed horse blood and, in the case of selective media, trimethoprim (5 μg/ml), vancomycin (10 μg/ml), nalidixic acid (20 μg/ml) and amphotericin B (5 μg/ml), were added. All the antibiotics were from Sigma-Aldrich Corporation (St. Louis, MO, USA). The agar plates were simultaneously incubated at 37°C under humidified conditions with 10% carbon dioxide. *H. pylori*-like colonies were confirmed by positivity with urease, oxidase and catalase tests.

### Multiplex Assay for Gut Hormones and Cytokines

Milliplex MAP Kit for Mouse Metabolic Magnetic Bead Panel (Catalogue# MMHMAG-44K; Millipore Corp., MO, USA) was used to quantify eight gut hormones that are important regulators of food intake, energy expenditure and body mass: acylated (active) ghrelin, leptin, active amylin, insulin, active glucagon-like peptide-1 (GLP-1), total gastric inhibitory polypeptide (GIP), total peptide YY (PYY) and pancreatic polypeptide (PP). Mouse Cytokine/Chemokine Magnetic Bead Panel (Catalogue# MPXMCYTO-70K; Millipore Corp., MO, USA) was used to measure a panel of 32 different mouse cytokines/chemokines. Plasma was used and assays were carried out according to manufacturer's instructions. Measurement was performed on Luminex 200™ system with xPONENT™ software, version 3.1 (Luminex, Austin, TX, USA).

### Statistical analysis

Statistical analyses were performed using the IBM SPSS version 22.0 software. One-way ANOVA (alpha = 0.05) and Tukey's honestly significant difference (HSD) post-hoc test were performed. Parametric Pearson correlation was carried out. P-value of < 0.05 was considered significant.

## Author Contributions

Y.K., W.Y.W., Q.H.P. and I.M.D.S.: Carried out the lab investigation and data analysis. S.W.S., J.O., A.A.A. and R.M.B.: Animal study. S.P., M.F.L., J.V., K.H.C. and T.L.T.: Planned the experiments.

## Figures and Tables

**Table 1:** Parametric Pearson correlation between levels of hormones related to energy homeostasis/chemokines and body mass accompanied by two-tailed p-values

	8 Weeks		16 Weeks	
	Correlation Coefficient	Significance (2-tailed)	Correlation Coefficient	Significance (2-tailed)
**Ghrelin**	−0.344	0.364	−0.803*	0.016
**Leptin**	0.724*	0.027	0.788*	0.020
**Insulin**	0.586	0.098	0.838**	0.009
**PYY**	−0.732*	0.025	−0.873**	0.005
**Eotaxin**	0.787*	0.012	0.431	0.286

*. Correlation is significant at the 0.05 level (2-tailed).

**. Correlation is significant at the 0.01 level (2-tailed).
